# Clinical Characteristics and Current Treatment Modality of Preterm Infants with *Ureaplasma* spp. Infection

**DOI:** 10.3390/children11101202

**Published:** 2024-09-30

**Authors:** Zhenhai Zhang, Jian Wang, Wenwen Chen, Liping Xu

**Affiliations:** Zhangzhou Affiliated Hospital, Fujian Medical University, Zhangzhou 363000, China; zhenhai0596@126.com (Z.Z.); zzsyywangjian@163.com (J.W.); pipixiu@163.com (W.C.)

**Keywords:** *Ureaplasma* spp., preterm birth, mechanical ventilation, white blood cell count, bronchopulmonary dysplasia

## Abstract

Background: The impact of and countermeasures for *Ureaplasma* spp. in neonates remain controversial. The aim of this study was to evaluate the associated perinatal factors that can predict the likelihood of respiratory tract *Ureaplasma* spp. colonization and analyze the subsequent clinical course of affected infants, thereby providing the rationale for their diagnosis, treatment, and future study. Methods: This was a retrospective observational study of infants born at a gestational age (GA) of less than 32 weeks. Results: The prevalence of respiratory tract *Ureaplasma* spp. colonization was 25.8% (75/291), and it increased with a decrease in GA and birth weight (BW). Maternal vaginal *Ureaplasma* spp. colonization increased the risk of neonatal *Ureaplasma* spp. colonization, with an OR of 7.8 (95% CI: 3.1, 20.0). Infants with *Ureaplasma* spp. colonization had a higher white blood cell (WBC) count, normal C-reactive protein (CRP) level, and higher failure rate of weaning from mechanical ventilation (30.7% vs. 17.1%, *p* = 0.014); they also suffered more from interstitial pneumonia (20.0% vs. 5.6%, *p* < 0.001) and bronchopulmonary dysplasia (36.0% vs. 13.4%, *p* < 0.001). Infants receiving anti-*Ureaplasma* spp. treatment had a lower GA, lower BW, and more severe respiratory syndromes. However, the difference in respiratory manifestation became insignificant after adjusting for GA. Conclusions: GA and maternal vaginal *Ureaplasma* spp. colonization could be used to predict neonatal respiratory tract *Ureaplasma* spp. colonization. An elevated WBC count combined with normal CRP is a good marker of *Ureaplasma* spp. colonization/infection. It is conventional practice to start anti-*Ureaplasma* spp. treatment when infants present with a deteriorated respiratory condition. This practice warrants further investigation considering GA as a predominant intermediate variable.

## 1. Introduction

*Ureaplasma* species (*Ureaplasma* spp.) are the most common organisms present in the genital tract of pregnant women [1]. The invasion of the amniotic cavity by *Ureaplasma* spp. can cause chorioamnionitis, fetal inflammatory response syndrome, and preterm birth (PTB) [2], as demonstrated by the isolation of *Ureaplasma* spp. in placentae and amniotic fluid [3,4,5]. *Ureaplasma* spp. are also frequently isolated from the respiratory tract of preterm infants through vertical transmission from their mothers [6,7]. Colonization or infection with *Ureaplasma* spp. has been associated with a series of neonatal morbidity and sequelae, especially chronic lung disease (CLD) and bronchopulmonary dysplasia (BPD) [8]. With the increasing survival of the more immature infants, BPD continues to be one of the most hazardous health-threatening complications and places a substantial burden on families and the public. Numerous studies have shown that *Ureaplasma* spp. can induce a pro-inflammatory and pro-fibrotic response, thus contributing to interstitial pneumonia and the development of BPD [9,10,11,12]. However, some studies found that positive *Ureaplasma* spp. screening alone was not associated with BPD [13]. A recent meta-analysis did not draw a conclusion about the role of *Ureaplasma* spp. in adverse pregnancy and birth outcomes [14]. The heterogeneity of these studies might be attributed to “small study effects” or underlying confounding factors [15,16].

Since the pathogenic effect of *Ureaplasma* spp. on neonatal respiratory outcomes is controversial, there is still no consensus on which antimicrobial modality should be used to counteract *Ureaplasma* spp. colonization or infection. As opportunistic pathogens, it is difficult to clearly define their colonization or infection in a newborn infant. Diagnosing *Ureaplasma* spp. infection remains a challenge. Conventionally, *Ureaplasma* spp. confirmed either by culture or RT-PCR from tracheal or nasopharyngeal secretions is thought of as an infection [17,18]. Only a few studies have investigated the specific clinical course of *Ureaplasma* spp. infection [19]. Early identification of infants at the highest risk of *Ureaplasma* spp. infection would help to initiate appropriate treatments. Herein, we conducted this study with the aim of evaluating relevant perinatal factors that could predict the likelihood of respiratory tract *Ureaplasma* spp. colonization and analyzing the subsequent clinical course of affected infants; in doing so, we hoped to provide a rationale for their diagnosis, treatment, and future study.

## 2. Materials and Methods

### 2.1. Subjects

This study included all infants who were born before a gestational age (GA) of 32 weeks and admitted to the neonatal intensive care unit (NICU) at Zhangzhou Affiliated Hospital of Fujian Medical University in China between January 2022 and December 2023. The exclusion criteria were as follows: (1) infants with major congenital malformations; (2) infants who died within the first 7 days after birth; (3) infants who were transferred to other hospitals before 36 weeks postmenstrual age (PMA); and (4) infants who were unable to continue treatment before 36 PMA due to parental wishes.

Data were extracted from the electronic medical record system, including demographic information, obstetric complications, and neonatal morbidities.

### 2.2. Clinical Practice for Ureaplasma spp. Detection

In our NICU, preterm infants were routinely screened for *Ureaplasma* spp. upon admission. Respiratory tract samples were obtained from the tracheal tubes (tracheal aspirates) or nasopharynx (swabs or sputum). Both nasopharyngeal and tracheal samples could be used for detection, as described previously [20]. Pregnant women who had signs of premature birth selectively underwent examination for Ureaplasma spp. using vaginal swabs. Swabs were infiltrated with saline upon collection. Swabs, aspirates, and sputum were placed in sterile and sealed containers for examination [21]. DNA was extracted using a magnetic bead-based method on an automatic extractor (GeneRotex 96) before being amplified and analyzed on an RT-PCR platform (Abi7500) in accordance with the manufacturer’s instructions. The PCR detection kit was specific for *Ureaplasma* spp. but could not differentiate between biovars and serovars. We used an existing detection protocol outlined in previous studies [19].

### 2.3. Clinical Practice for CBC and CRP Detection

All infants had two consecutive complete blood count (CBC) and C-reactive protein (CRP) examinations (in a 24 h interval) after birth.

### 2.4. The Initial Administration of Antibiotics

All enrolled infants received intravenous penicillin (50,000 units·kg^−1^) plus cefotaxime (50 mg·kg^−1^) every 12 h for at least 72 h until the results of cultures were available.

### 2.5. Diagnostic Criteria

PTB is a heterogeneous syndrome consisting of various phenotypes based on etiologic complexities [22]. In this study, we divided PTB into two subgroups known as “Spontaneous PTB” and “Iatrogenic PTB”.

Spontaneous PTB: preterm birth that follows spontaneous labor or premature rupture of the membranes (PROM) [23].

Iatrogenic PTB: preterm birth initiated by the provider of maternity care in response to maternal illness or signs of fetal compromise, including placental abruption, pre-existing hypertension, pre-eclampsia, antepartum hemorrhage, fetal distress, etc. [24].

Interstitial pneumonia was diagnosed depending on the appearance of chest radiographs, encompassing diffuse streaky interstitial infiltration and coarse reticular infiltration [25]. All infants received a chest radiographic examination within 24 h after birth and at least one more chest radiographic examination before 36 weeks PMA.

BPD was defined as the need for supplemental oxygen or assisted ventilation (including continuous positive airway pressure, intermittent positive pressure ventilation, nasal continuous positive airway pressure, non-invasive positive pressure ventilation, and nasal cannula flow) for ≥3 consecutive days to maintain arterial oxygen saturation in the 90–95% range at 36 weeks PMA [26].

Patent ductus arteriosus (PDA) was diagnosed if echocardiography showed signs of ductus arteriosus shunt. All infants received a bedside echocardiographic examination within 7 days of birth.

### 2.6. Ethics

This was a retrospective study. Approval for this study was obtained from the Institutional Review Board of Zhangzhou Affiliated Hospital of Fujian Medical University (2022KYZ297); informed consent was waived due to the retrospective nature of the study.

### 2.7. Statistical Analysis

Statistical analysis was performed using SPSS 26.0 software. The Chi-square test or Fisher’s exact test was used to analyze categorical variables. Continuous data with a normal distribution are described as means ± SDs and were analyzed using a *t*-test of two independent samples between the two groups. Continuous data that did not conform to a normal distribution are presented as the medians (25% percentiles, 75% percentiles) and were analyzed among groups using the Mann–Whitney U test. Multivariate regression was used to calculate the adjusted advantage ratio and the corresponding 95% confidence interval. A double-tailed *p*-value of <0.05 was considered statistically significant.

## 3. Results

### 3.1. Prevalence of Ureaplasma spp. in Preterm Infants Born before 32 Weeks GA

**Figure 1:**
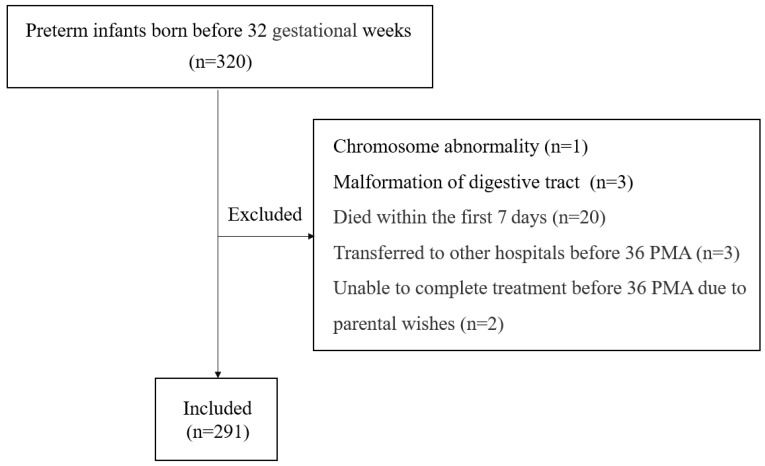
Flow diagram of the study.

A total of 320 preterm infants born before a GA of 32 weeks were admitted to our NICU during the study period. Four infants had severe deformities. Twenty infants died within the first 7 days after birth. Before 36 weeks of PMA, three infants were transferred to other hospitals, and two infants were unable to continue treatment due to parental wishes. Thus, a final 291 infants were eligible for the analysis (Figure 1). The mean GA was 29.1 ± 1.8 weeks (range: 23 weeks +5 days–31 weeks +6 days), and the mean birth weight (BW) was 1335 g (range: 540–2300 g). Among the study population, 85.9% (250/291) were defined as spontaneous PTBs and 14.1% (41/291) were defined as iatrogenic PTBs. The proportion of in vitro fertilization (IVF) was 8.6% (25/291). All the included infants were tested for *Ureaplasma* spp., and the overall positive rate was 25.8% (75/291). The rate of positivity was similar in tracheal tube samples (28.8%) and nasopharyngeal samples (23.5%). The rate of positivity reached 35.9% among neonates with GA < 30 weeks and 51.8% among neonates with GA < 28 weeks. With the decrease in GA, the rate of positivity increased (Table 1). The odds ratio (OR) of every one week decrease in GA to *Ureaplasma* spp. colonization was 1.5 (95% CI: 1.3–1.7, *p* < 0.001). In addition to GA, the rate of positivity was also higher among infants with lower BW. *Ureaplasma* spp. was detected in 37.5% of neonates weighing <1250 g and 50.0% of neonates weighing <1000 g.

A total of 164 pregnant women were tested for *Ureaplasma* spp. before delivery. The prevalence of *Ureaplasma* spp. colonization in the maternal vaginal tract was 52.4% (i.e., 86 of 164 samples collected before delivery). Among spontaneous PTBs, 79 individuals tested positive. The rate of positivity was higher in spontaneous PTBs than in iatrogenic PTBs (55.6% vs. 31.8%, *p* = 0.037). There was no difference in maternal *Ureaplasma* spp. colonization between women who underwent IVF and women who conceived spontaneously (63.2% vs. 51.0%, *p* = 0.320).

### 3.2. Perinatal Factors Associated with Neonatal Ureaplasma spp. Colonization

Infants with *Ureaplasma* spp. colonization were born at an earlier GA and had a lower BW than those without *Ureaplasma* spp. colonization. A higher proportion of spontaneous PTB, PROM > 18 h, maternal vaginal *Ureaplasma* spp. colonization and vaginal delivery were observed in mothers who delivered infants colonized by *Ureaplasma* spp. (Table 2). The rate of each form of conception (IVF versus spontaneous) did not differ significantly between *Ureaplasma* spp.-positive and -negative groups. After adjusting for GA via logistic regression, maternal vaginal *Ureaplasma* spp. colonization was still significantly associated with neonatal *Ureaplasma* spp. colonization (Table 3).

### 3.3. Postnatal Clinical Characteristics of Neonatal Ureaplasma spp. Colonization

The consecutive monitoring of blood tests after birth showed that the white blood cell (WBC) count of infants with *Ureaplasma* spp. colonization was higher than that of infants without *Ureaplasma* spp. colonization, while the CRP levels did not differ significantly between the two groups. Further comparison between different GA groups showed that an increase in WBC count occurred in subgroups divided by GA (Table 4). In addition to blood index, their clinical manifestation showed that infants with *Ureaplasma* spp. colonization were more frequently ventilated within 72 h after birth, and the rates of interstitial pneumonia and BPD were higher than that in the *Ureaplasma* spp.-negative group (Table 2). However, after adjusting for GA via logistic regression, the differences were not significant, indicating that GA might be a prominent confounding variable (Table 3).

Of the 75 infants with *Ureaplasma* spp. colonization, 50 (66.7%) infants received treatment with erythromycin (35 infants—15 mg·kg^−1^ every 12 h for 14 days) or azithromycin (15 infants—10 mg·kg^−1^ for 7 days followed by 5 mg·kg^−1^ for 7 days). Compared with infants who did not receive drug treatment, infants in the treatment group had lower GA and BW and a higher WBC count (Table 5). Higher rates of failure to respond to non-invasive respiratory support within 72 h, interstitial pneumonia, and BPD were also observed in the treatment group. These results revealed that neonatologists tended to give prescriptions to infants based on GA, BW, WBC count, and the deterioration of the respiratory condition. However, after stratification by GA, the difference in respiratory manifestation became insignificant (Table 6).

Among the 50 infants who received drug treatment, the rate of BPD did not differ significantly between those who received erythromycin versus azithromycin (erythromycin group: 40.0%; azithromycin group: 53.3%, *p* = 0.384).

## 4. Discussion

This study revealed the epidemiological characteristics of *Ureaplasma* spp. colonization in pregnant women and their offspring with a GA of less than 32 weeks. The overall prevalence of *Ureaplasma* spp. colonization was 52.4% in the maternal vaginal tract and 25.8% in the neonatal respiratory tract, respectively. Maternal vaginal *Ureaplasma* spp. colonization was consistently associated with neonatal respiratory tract *Ureaplasma* spp. colonization. Sobouti B et al. reported a rate of transmission from mothers to their neonates as high as 60–70% [27]. Screening of mothers would help to identify the risk of neonatal infection. If a vaginal test is unfeasible, perinatal factors—such as spontaneous PTB, PROM, and vaginal delivery—could be used for tentative prediction because these factors were also partially associated with neonatal *Ureaplasma* spp. colonization in the compound GA strata. PTB is a heterogeneous syndrome encompassing various phenotypes based on etiology [22]. It is well-recognized that two of its major clinical etiologies are iatrogenic and spontaneous PTB [28]. There is growing awareness that PTB phenotypes are associated with neonatal outcomes; this has prompted earlier prenatal interventions with the aim of reducing perinatal complications [29]. Spontaneous PTBs account for the majority of PTBs, and their cause has remained imperceptible. In many cases, spontaneous PTBs are thought to be caused by infection or inflammatory processes [30]. Among the pathogenic bacteria associated with chorioamnionitis, *Ureaplasma* spp. are the most prevalent organisms isolated from placental membranes and amniotic fluid [31,32,33]. Gerber S et al. tested *Ureaplasma* spp. in transabdominal amniotic fluid among asymptomatic women at 15–17 weeks gestation and found that *Ureaplasma* spp.-positive women were at greater risk of subsequent preterm labor and delivery [34]. Some scholars have suggested placing *Ureaplasma* spp. within the context of chorioamnionitis diagnosis. Prior to intrauterine infection, lower genital tract *Ureaplasma* colonization is suspected [35,36]. Its consequences might depend on the virulence, bacterial load, duration, and host immune response [37]. Previous studies have demonstrated that colonization of *Ureaplasma* spp. in the female vagina could activate the production of cytokines, prostaglandins, uterine contractions, and dilatation of the cervix, causing spontaneous PTB with an intact membrane or PROM [38,39]. PROM has historically been classed as spontaneous PTB [28]. Maternal *Ureaplasma* spp. infection mainly occurs in pregnancies after PROM, which might increase the likelihood of vertical transmission to the fetus or neonate [36]. Viscardi RM et al. demonstrated that PROM > 72 h is a good predictor for *Ureaplasma* spp. lower airway tract colonization in preterm infants [18]. A shorter duration of PROM (PROM >18 h) was found to be significant in our study. This result indicated the necessity of targeted monitoring and intervention in the early stages of PROM.

In this study, we found that there was a strong inverse association between neonatal *Ureaplasma* spp. colonization and GA. As GA decreased to less than 28 weeks, the prevalence of *Ureaplasma* spp. reached 51.5%. Sung et al. reported a higher rate (65%) as GA decreased to less than 26 weeks [7]. We further determined that for every week of decline in GA, the risk of *Ureaplasma* spp. colonization increased. A similar association was observed between Ureaplasma spp. colonization and BW. These results were consistent with previous studies [21]. Ozdemir et al. reported a positive *Ureaplasma* spp. detection rate of 33.0% among neonates weighing < 1250 g [40]. A similar rate (37.5%) was reported in our study, and we found a higher rate (50.0%) among infants weighing < 1000 g. Since GA and BW have a strong linear correlation, including GA in logistic regression is customary in observational studies. After incorporating candidate factors, the risk of *Ureaplasma* spp. respiratory tract colonization in infants can be simply identified antenatally via GA and maternal vaginal *Ureaplasma* spp. colonization.

The WBC count, shortly after birth, of infants colonized by *Ureaplasma* spp. was found to be significantly elevated in our study. A similar result has been found in previous studies [17,41]. It is well known that elevated WBC count is a hematologic change indicative of systemic inflammatory response syndrome (SIRS) [42]. Local inflammation characterized by an increase in chemotactic activity and neutrophil count was also found in the tracheobronchial aspirate of patients from the *Ureaplasma* spp.-colonized group, confirming that *Ureaplasma* spp. can trigger an inflammatory response [43]. However, an elevated WBC count is often seen in early-onset sepsis (EOS). Another thoroughly investigated laboratory marker used to diagnose neonatal sepsis is CRP [44]. We found that although the WBC count increased in the *Ureaplasma* spp. group, CRP did not increase simultaneously, which was consistent with the findings revealed by Meadows JT et al. [45]. A combination of increased WBC count and negative CRP might be helpful in distinguishing *Ureaplasma* spp. infection from bacterial EOS, since CRP always appears increased upon consecutive monitoring of the latter [46]. Regarding short-term neonatal outcomes, several studies have found that infants with *Ureaplasma* spp. respiratory colonization had a higher incidence of respiratory distress syndrome (RDS), interstitial pneumonia, and BPD [20,47,48]. In our study, in infants with *Ureaplasma* spp. colonization, we observed a higher rate of failure of weaning from mechanical ventilation within 72 h of birth, interstitial pneumonia, and BPD. However, these results must be interpreted with caution since after adjusting for GA, we failed to detect any differences. A reasonable explanation for this is that the association of *Ureaplasma* spp. with neonatal respiratory outcomes might be overestimated thanks to the weighted contribution of GA. Therefore, in the future, the real effects of *Ureaplasma* spp. on preterm outcomes must be analyzed alongside a more detailed GA stratification.

A growing number of scholars have observed that the detection of *Ureaplasma* spp. in the respiratory tract is not solely indicative of real infection, which places the treatment in a dilemma. As there are no guidelines for clinicians to follow, the decision is always made based on personal empirical judgment, resulting in variations in practice across NICUs. Generally speaking, treatments are informed by a suspected *Ureaplasma* spp. infection rather than colonization. In our NICU, infants who received treatment had a cluster of characteristics including lower GA, lower BW, higher WBC count, more dependence on mechanical ventilation after 72 h of life, chest X-ray images indicative of interstitial pneumonia, and a higher risk for BPD, suggesting that clinicians largely treat infants who require intensive respiratory support and suffer with poor respiratory conditions. Theilen U et al. also found an increasing trend in interstitial changes (as found via radiology) and a prolonged course of ventilation in infants with *Ureaplasma* spp. in their tracheal secretion [48]. Infants who underwent prolonged ventilation were at high risk of BPD [49] and, therefore, became potential receivers of anti-*Ureaplasma* spp. treatment. However, their poor respiratory conditions might be attributed to their more immature GA. It is urgent to investigate specific biomarkers that can guide clinical practice in the first few hours of life. Based on the retrospective nature of this study and the greater immaturity of the treated group at baseline, we cannot draw a conclusion on the benefit of an antimicrobial regimen. The predominant drugs used in treatment for neonates with *Ureaplasma* spp. infection are erythromycin and azithromycin. Previous randomized trials have found that erythromycin could not eliminate *Ureaplasma* spp. from the airways and failed to reduce the incidence of chronic lung disease [50,51]. Viscardi RM et al. found that a 3-day azithromycin regimen (20 mg·kg^−1^ every 24 h for 3 days) effectively eradicated respiratory tract *Ureaplasma* colonization but failed to decrease the risk of BPD [52]. However, in recent studies, Chen X et al. found that a 2-week effective azithromycin treatment (10 mg·kg^−1^ for 7 days followed by 5 mg·kg^−1^ for 7 days) in *Ureaplasma* spp.-positive VLBW infants were associated with a reduced risk of BPD [19], and Ballard HO et al. pointed out that early treatment of *Ureaplasma*-colonized/infected infants with azithromycin might decrease the occurrence of BPD and death [53]. After all, most scholars consider azithromycin the superior choice for anti-*Ureaplasma* spp. treatment. Recently, azithromycin therapy (a 10-day course of intravenous azithromycin 20 mg·kg^−1^ for 3 days, followed by 10 mg·kg^−1^ for 7 days) for CLD secondary to prematurity in the AZTEC study failed to induce a decline in moderate or severe CLD [54]. However, in the AZTEC trial, azithromycin was administrated prophylactically, regardless of *Ureaplasma* spp. colonization. Infants with respiratory tract *Ureaplasma* spp. colonization should be targeted; in doing so, we will accrue more evidence to support clinical management. In addition to azithromycin and erythromycin, Motomura K et al. found that clarithromycin could reduce adverse pregnancy and neonatal outcomes induced by *Ureaplasma* spp. [55]. This regimen should be assessed for efficacy and safety in future RCTs.

This is a retrospective study containing intact data on *Ureaplasma* spp. detection and the basic demographic information of pregnant women and their offspring, which facilitate reliable analysis of the perinatal epidemiological characteristics of *Ureaplasma* spp. The results herein provide a basis for further investigation of optimal biomarkers and treatment indications when GA is considered as an intermediate or confounding variable. However, this study has some limitations: Firstly, we did not distinguish between biovars or serotypes of *Ureaplasma* spp. Several studies have found that *Ureaplasma. parvum* is more pathogenic in respiratory disease [20]. Evidence of the differences in virulence between serovars is scarce [7]. Secondly, in the absence of relevant data, we did not analyze coinfections with other pathogens or complications, such as bacterial vaginitis and chorioamnionitis. Thirdly, since our sample size was relatively small, our categorization of PTB did not allow us to differentiate between more detailed groups according to an updated taxonomy [22].

## 5. Conclusions

GA and maternal vaginal *Ureaplasma* spp. colonization could be used to predict neonatal respiratory tract *Ureaplasma* spp. colonization. An elevated WBC count combined with normal CRP is a good marker of *Ureaplasma* spp. colonization/infection. It is conventional practice to start anti-*Ureaplasma* spp. treatment when infants present with a deteriorated respiratory condition. This practice warrants further investigation considering GA as a predominant intermediate variable.

## Figures and Tables

**Table 1:** The prevalence of *Ureaplasma* spp. colonization in different GA groups.

GA (Weeks)	*n*	UU (n)	UU (%)
23 weeks + 5 days − 25 weeks + 6 days	13	10	76.9
26–26 weeks + 6 days	15	7	46.7
27–27 weeks + 6 days	28	12	42.9
28–28 weeks + 6 days	39	11	28.2
29–29 weeks + 6 days	50	12	24.0
30–30 weeks + 6 days	65	10	15.4
31–31 weeks + 6 days	81	13	16.0
Total	291	75	25.8

GA: gestational age, UU: *Ureaplasma* spp. infection.

**Table 2:** Clinical characteristics of the study population.

	UU	Non-UU	*p*
GA (weeks)	28.7 ± 2.1	29.8 ± 1.6	0.001
BW (g)	1182 ± 344	1388 ± 315	<0.001
IVF % (n)	6.7 (5/75)	9.3 (20/216)	0.490
Spontaneous PTB % (n)	93.3 (70/75)	83.3 (180/216)	0.032
PROM % (n)	45.3 (34/75)	25.9 (56/216)	0.002
Maternal UU % (n)	82.1 (46/56)	37.0 (40/108)	<0.001
Vaginal delivery % (n)	64.0 (48/75)	44.0 (95/216)	0.003
First WBC count (counts/μL)	13,200 (8400, 20,900)	6800 (5000, 9200)	<0.001
Second WBC count (counts/μL)	18,600 (10,900, 32,100)	8000 (6000, 12,000)	<0.001
irst CRP (mg/L)	3.0 (1.1, 7.6)	3.3 (1.1, 11.9)	0.473
Second CRP (mg/L)	2.2 (0.6, 7.7)	1.8 (0.6, 6.0)	0.629
PDA % (n)	44.0 (33/75)	46.8 (101/216)	0.680
Failed to respond to non-invasive respiratory support within 72 h % (n)	30.7 (23/75)	17.1 (37/216)	0.014
Interstitial pneumonia % (n)	20.0 (15/75)	5.6 (12/216)	<0.001
Lung consolidation % (n)	12.0 (9/75)	8.8 (19/216)	0.418
BPD % (n)	36.0 (27/75)	13.4 (29/216)	<0.001

GA: gestational age, BW: birth weight, PTB: preterm birth, IVF: in vitro fertilization, PTB: preterm birth, UU: *Ureaplasma* spp.-positive, PROM: premature rupture of fetal membranes > 18 h, WBC: white blood count, CRP: C-reactive protein, PDA: patent ductus arteriosus, BPD: bronchopulmonary dysplasia.

**Table 3:** Association between perinatal factors and neonatal *Ureaplasma* spp. colonization.

	OR [95% CI]	aOR [95% CI]
Spontaneous PTB	2.8 [1.1, 7.4]	1.2 [0.4, 3.8]
	*p* = 0.039	*p* = 0.695
PROM	2.4 [1.4, 4.1]	1.9 [1.0, 3.8]
	*p* = 0.002	*p* = 0.069
Maternal UU	11.8 [5.0, 28.0]	7.8 [3.1, 20.0]
	*p* < 0.001	*p* < 0.001
Vaginal delivery	2.3 [1.3, 3.9]	1.9 [1.0, 3.6]
	*p* = 0.003	*p* = 0.068

PTB: preterm birth, PROM: premature rupture of the membranes, UU: *Ureaplasma* spp.-positive, aOR: adjusted for gestational age.

**Table 4:** Comparison of BW, WBC count, and serum CRP in different GA groups.

	GA < 28 Weeks			GA ≥ 28 Weeks		
	UU n = 29	Non-UU n = 27	*p*	UU n = 46	Non-UU n = 189	*p*
BW (g)	855 ± 174	915 ± 189	0.224	1388 ± 252	1455 ± 268	0.124
First WBC count (counts/μL)	17,420 (9355, 27,890)	5450 (4160, 8280)	<0001	12,105 (6330, 16,140)	6850 (5050, 9260)	<0001
Second WBC count (counts/μL)	25,740 (12,560, 49,090)	11,950 (6270, 17,110)	<0001	14, 485 (9913, 21,838)	7900 (5800, 11,605)	<0001
First CRP (mg/L)	3.4 (1.3, 11.3)	8.7 (3.2, 19.4)	0.065	2.1 (0.8, 7.0)	2.9 (1.1, 10.4)	0.379
Second CRP (mg/L)	4.4 (1.0, 9.0)	6.5 (2.3, 21.6)	0.241	1.4 (0.5, 4.7)	1.3 (0.6, 4.3)	0.550

GA: gestational age, BW: birth weight, UU: *Ureaplasma* spp.-positive, WBC: white blood count, CRP: C-reactive protein.

**Table 5:** Clinical characteristics of infants with and without anti-*Ureaplasma* spp. treatment.

	Treated	Untreated	*p*
GA (weeks)	27.6 ± 2.0	29.2 ± 1.9	0.002
BW (g)	1097 ± 293	1353 ± 379	0.002
First WBC count (counts/μL)	15,320 (9428, 25,127)	8850 (5560, 11,425)	0.001
Second WBC count (counts/μL)	22,775 (11,568, 34,743)	11,430 (9960, 18,895)	0.003
Failed to respond to non-invasive respiratory support within 72 h %(n)	42.0 (21/50)	8.0 (2/25)	0.003
Interstitial pneumonia %(n)	28.0 (14/50)	4.0 (1/25)	0.014
BPD %(n)	44.0 (22/50)	20.0 (5/25)	0.041

GA: gestational age, BW: birth weight, WBC: white blood count, BPD: bronchopulmonary dysplasia.

**Table 6:** Clinical characteristics of infants with and without anti-*Ureaplasma* spp. treatment in different GA groups.

	GA < 28 Weeks			GA ≥ 28 Weeks		
	Treated	Untreated	*p*	Treated	Untreated	*p*
BW	857 ± 150	843 ± 323	0.934	1336 ± 183	1450 ± 308	0.146
Failed to respond to non-invasive respiratory support within 72 h %(n)	64.0 (16/25)	50.0 (2/4)	1.000	20.0 (5/25)	0 (0/21)	0.090
Interstitial pneumonia %(n)	44.0 (11/25)	25.0 (1/4)	0.865	12.0 (3/25)	0 (0/21)	0.297
BPD %(n)	80.0 (20/25)	75.0 (3/4)	1.000	8.0 (2/25)	9.5 (2/21)	1.000

GA: gestational age, BW: birth weight, WBC: white blood count, BPD: bronchopulmonary dysplasia.

## Data Availability

The datasets used and/or analyzed during the current study are available from the corresponding author upon reasonable request, due to privacy.

## Abbreviations

NICU	Neonatal intensive care unit
PMA	Postmenstrual age
PTB	Preterm birth
CLD	Chronic lung disease
BPD	Bronchopulmonary dysplasia
PDA	Patent ductus arteriosus
UU	*Ureaplasma* spp.
PROM	Premature rupture of fetal membrane
IVF	in vitro fertilization
GA	Gestational age
BW	Birth weight
CBC	Complete blood count
WBC	White blood count
RDS	Respiratory distress syndrome
CRP	C-reactive protein
EOS	Early-onset sepsis
SIRS	systemic inflammatory response syndrome
VLBW	Very low birth weight
